# Vasculature Remodeling in a Rat Model of Cerebral Ischemia. The Fate of the BrdU-Labeled Cells Prior to Stroke

**DOI:** 10.3389/fneur.2018.01014

**Published:** 2018-11-27

**Authors:** Roxana Surugiu, Daniela Glavan, Mircea Popescu, Otilia Margaritescu, Radu Eugen, Aurel Popa-Wagner

**Affiliations:** ^1^Center of Clinical and Experimental Medicine, University of Medicine and Pharmacy of Craiova, Craiova, Romania; ^2^Psychiatry Clinic Hospital, University of Medicine and Pharmacy of Craiova, Craiova, Romania; ^3^Department of Neurosurgery, University of Medicine and Pharmacy of Craiova, Craiova, Romania; ^4^Molecular Biology and Pathology Research Lab, University Hospital Bucharest, Bucharest, Romania; ^5^Griffith University School of Medicine, Southport, QLD, Australia

**Keywords:** angiogenesis, stroke, BrdU-labeling, endothelial cells, blood vessel remodeling

## Abstract

Despite the clinical significance of post-stroke angiogenesis, a detailed phenotypic analysis of pre-stroke vascular remodeling and post-stroke angiogenesis had not yet been done in a model of focal ischemia. In this study, using BrdU-labeling of proliferating cells and immunofluorescence of pre- and post-stroke rats, we found that, (i) BrdU administered before stroke was incorporated preferentially into the nuclei of endothelial cells lining the lumen of existing blood vessels and newly born neurons in the dentate gyrus but not in the subventricular zone or proliferating microglia, (ii) BrdU injection prior to stroke led to the patchy distribution of the newly incorporated endothelial cells into existing blood vessels of the adult rat brain, (iii) BrdU injection prior to stroke specifically labeled neuronal precursors cells in a region of soft tissue beyond the inhibitory scar, which seems to be permissive to regenerative events, (iv) BrdU injection after stroke led to labeling of endothelial cells crossing or detaching from the disintegrating blood vessels and their incorporation into new blood vessels in the stroke region, scar tissue and the region beyond, (v) BrdU injection after stroke led to specific incorporation of BrdU-positive nuclei into the “pinwheel” architecture of the ventricular epithelium, (vi) blood vessels in remote areas relative to the infarct core and in the contralateral non-lesioned cortex, showed co-labeled BrdU/RECA^+^ endothelial cells shortly after the BrdU injection, which strongly suggests a bone marrow origin of the endothelial cells. In the damaged cortex, a BrdU/prolyl 4-hydroxylase beta double labeling in the close proximity to collagen IV-labeled basement membrane, suggests that, in addition to bone marrow derived endothelial cells, the disintegrating vascular wall itself could also be a source of proliferating endothelial cells, (vii) By day 28 after stroke, new blood vessels were observed in the perilesional area and the scar tissue region, which is generally considered to be resistant to regenerative events. Finally, (viii) vigorous angiogenesis was also detected in a region of soft tissue, also called “islet of regeneration,” located next to the inhibitory scar.

**Conclusion:** BrdU administered prior to, and after stroke, allows to investigate brain vasculature remodeling in the adult brain.

## Introduction

Following recanalization and neuroprotection, research on stroke is focused on tissue structure restoration and functional recovery based on revascularization and cell therapy based neuroregeneration. Angiogenesis is most likely the first step in supporting endogenous recovery mechanisms, like neurogenesis in the subventricular zone and perilesional area and is promoted by several signaling molecules and growth factors, such as eNOS and CSE, VEGF/VEGFR2, Ang-1/Tie2, IGF-1, BDNF, FGF-2, VEGF, and the chemokines SDF-1 and MCP-1 (1). However, the molecular and genetic events underlying successful angiogenesis are not fully understood and therefore cannot be exploited for stroke therapy (2–5).

Endogenous recovery and long-term post-stroke repair mechanisms in the brain rely on angiogenesis, which can be enhanced by release of pro-angiogenic factors in response to, for example, physical exercise in the brain. Thus, exposure of Wistar rats to treadmill training increased the expression of vascular endothelial growth factor (VEGF) and matrix metalloproteinase 2 (MMP2) (6).

Few studies have investigated human post-stroke angiogenesis at molecular level. Krupinski and colleagues (7) noted active angiogenesis in the penumbral areas of patients who survived from several days to weeks after cerebral stroke, along with a positive correlation between microvessel density and patient survival. In subsequent studies, the authors demonstrated an increased synthesis of angiogenic growth factors, such as FGF-2, PDGF, VEGF, and their receptors within hours of stroke that correlated with blood vessel growth in the penumbra (8, 9).

The literature on gene expression profiles after stroke in humans and rodents is limited. Recently, by comparative transcriptomics of angiogenesis in young vs. aged rats, our group identified 36 new stroke-related genes which may be used to develop new therapeutic approaches to improve angiogenesis after stroke in the aged brain (10). In another study, Vikman and Edvinsson (11) have shown similarities in gene expression profiles between human strokes and those in animal models and reported new genes that support the dynamic changes that occur after stroke in the middle cerebral artery branches supplying the ischemic region.

These results argue for the utility of proangiogenic therapies in stroke to support the restoration and recovery of neurovascular networks after ischemia by increasing blood flow and decreasing infarct size (5). However, there are parallels between the structure of the newly formed angiogenic blood vessels after stroke and blood vessels of cancerous tumors with regard to capillary immaturity and permeability which strongly suggest that the structure of the vascular wall in post-stroke angiogenesis is different from that of the non-injured, mature vascular wall. Therefore, in this study, we aimed at identifying the major contributors to vascular remodeling in naïve and post-stroke rats by making a detailed microscopic analysis of pre-stroke vascular remodeling and post-stroke angiogenesis focused on BrdU-labeled proliferating cells in pre- and post-stroke rats.

## Materials and methods

### Animals

**Figure 1:**
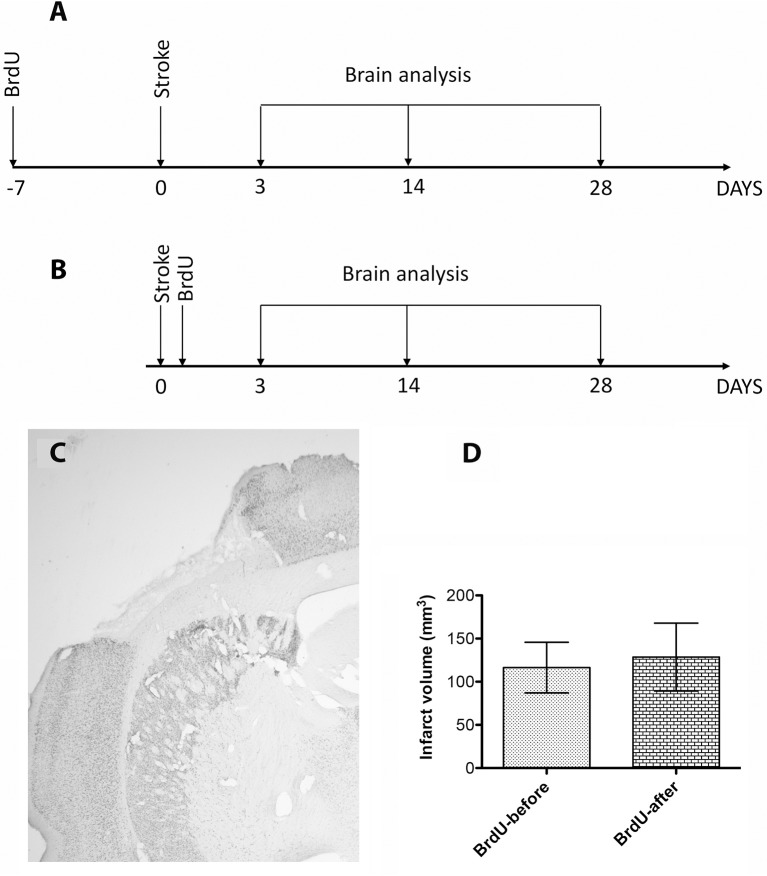
**(A,B)** Schematic overview of the experimental design. **(C)** The development of the infarct core by immunohistochemistry for NeuN. The infarct was larger (129 ± 39 mm^3^) in in the first 3 days, presumably because of edema buildup, and stabilized to 116 ± 29 mm^3^ by day 28 **(D)**. However, the infarcted cortical volumes were largely similar and independent of the BrdU treatment.

*Sixty five* young [3 to 5 month old] male Sprague-Dawley rats, bred in-house, were used. Body weights ranged from 290 to 360 g. The rats were kept in a controlled environment in standard cages at the temperature of 22°C, in the humidity level between 40 and 60%, and light period time range from 07.00 to 19.00 h. They had free access to food and water. The rats were divided randomly into 3, 14, and 28 day post-stroke survival groups (*N* = 20 per time point and treatment) and included post-stroke rats injected with BrdU 1 week before cerebral ischemia and sacrificed at days 3 (*N* = 10), at day 14 (*N* = 10) and 28 post-stroke (*N* = 10) as well as post-stroke rats injected with BrdU daily after stroke, sacrificed at days 3, 14, and 28 post-stroke (Figures 1A,B). In addition, 5 rats were used as sham controls. All experiments were approved by the University Animal Experimentation Ethics Board as meeting the ethical requirements of the University of Medicine and Pharmacy of Craiova, Romania.

### Reversible occlusion of the middle cerebral artery (MCAO)

Cerebral infarction was induced by transcranial interruption of blood flow by transiently lifting the middle cerebral artery with a tungsten hook as previously described (12). Eighteen hours prior to surgery, rats were deprived of food to minimize variability in ischemic damage that can result from varying plasma glucose levels. Water remained available at all times. The right middle cerebral artery (MCAO) was lifted with a tungsten hook attached to a micromanipulator (Maerzhaeuser Precision Micro-manipulator Systems, Fine Science Tools). Both common carotid arteries were then occluded by tightening pre-positioned thread loops for 90 min. Throughout surgery, anesthesia was maintained by spontaneous inhalation of 1–1.5% isoflurane in a mixture of 75% nitrous oxide and 25% oxygen. Body temperature was controlled at 37°C by a Homeothermic Blanket System (Harvard Apparatus). The local changes in blood flow were monitored using a laser Doppler device (Perimed, Stockholm, Sweden), and blood gases were measured at several time points during ischemia. A decrease in the laser Doppler signal to < 20% of control values was considered to indicate successful MCA occlusion. After 90 min, the common carotid arteries were re-opened. Surgery was performed under antiseptic conditions to minimize the risk of infection. Nevertheless, 3 rats died in the first week post-stroke. Subsequent to survival times of 3 or 28 days, rats were deeply anesthetized with 2.5% isoflurane, 75% nitrous oxide, and 25% oxygen, and the blood removed by perfusion with neutral buffered saline. Brains were cut into 2 mm slices and the periinfarcted area was microdissected under a microscope and stored at −70°C until use.

To study the phenotype of proliferating cells before stroke, rats were given injections of bromodeoxyuridine (BrdU; 50 mg/kg body weight, i.p.) daily and in total for 7 days. To label the newly generated cells after stroke, rats were given injections of bromodeoxyuridine (BrdU; 50 mg/kg body weight, i.p.) daily in the first week post-stroke.

### RNA extraction and RNA quality control

After the tissue was homogenized, total RNA was extracted from microdissected tissue using TRIzol reagent (Invitrogen Life Technologies, Karlsruhe, Germany). Genomic DNA was removed using the RNeasy Plus kit (Qiagen).

### Quantitative real-time PCR

For quantitative real time PCR (qPCR), we synthesized cDNA from total RNA with the High-Capacity cDNA reverse transcription kit (Applied Biosystems, USA). The qPCR was performed in 96-well 0.1-ml thin-wall PCR plates (Applied Biosystems) in the Step One Plus System (Applied Biosystems). Each 20 μl reaction contained 10 μl iQ SYBR Green Master Mix (BioRad Laboratories, Hercules, CA), 2 μl prolyl 4-hydroxylase beta (*P4H beta)* gene-specific forward and reverse primer mix (Qiagen, Alameda, CA), and 8 μl pre-diluted cDNA. Controls contained nuclease-free water instead of template primer. The cycling conditions were 3 min at 95°C to activate iTaq DNA polymerase, followed by 45 cycles with 30 s denaturation at 95°C, 30 s annealing at 58°C and 30 s elongation at 72°C. At the end of the amplification cycles, melting curves were used to validate PCR product specificity. All samples were amplified in triplicate. Data were analyzed using the ΔΔCt method (13). The expression levels of genes of interest were normalized to the average of expression level of the three housekeeping genes (GAPDH, HPRT1, and Ribosomal protein 19, RPL 19) from the same sample. So, the relative expression for a gene of interest was defined as the ratio of expression of the gene to that of the housekeeping gene. The fold change for *P4H beta* was defined as the ratio of the relative expression in the ipsilateral hemisphere (stroke lesioned, peri-infarcted or PI) to that in the naive animals. Primers were provided by Eurofinn, Germany.

### Determination of the infarct volume

To assess the size of the infarct induced by transient focal ischemia, every tenth section was stained with NeuN, a marker of neuronal nuclei. In previous studies, we have found that the disappearance of NeuN is a reliable indicator that neurons have been lost (14). Images of the stained sections were taken to cover the entire infarcted area, which was then calculated as the sum of partial areas using Image J analysis software. Integration of the resulting partial volumes gave the total volume of the ipsilateral hemisphere along with the total volume of the cortical infarct; lesion volume was then expressed as a percent of the hemispheric volume.

### Immunohistochemistry of rat tissue

Sections (25 μm-thick) were cut on a freezing microtome and processed for immunohistochemistry as previously described (15). Briefly, after incubation with blocking solutions containing 3% donkey serum/10 mmol/L PBS/0.3% Tween 20, tissue sections were exposed overnight at 4°C to mouse anti-NeuN (1:1000, Millipore, Germany) diluted in PBS containing 3% normal donkey serum and 0.3% Tween 20. After washing in PBS containing 0.3% Tween, the NeuN antigens were visualized by DAB staining.

### Laminin, SMA, BRDU triple labeling

Sections were incubated first with a mix of mouse anti-gamma smooth muscle actin (SMA) (1:1000, Sigma-Aldrich, Munich, Germany) and rabbit anti-laminin 5 (1:2000, Sigma, Munich, Germany) followed by a mix of donkey anti-mouse Cy3 and donkey anti-rabbit Cy2 to visualize both antigens. After a short fixation step with 4% paraformaldehyde, sections were subjected to the BrdU treatment. For BrdU detection, free-floating sections were incubated in 2 M HCl at 37°C for 30 min, and rinsed in 0.1 M borate buffer (pH 8.5) at room temperature for 10 min. After neutralization, sections were incubated in blocking solution containing 10% goat serum, 0.3% Triton X-100, 0.2% gelatin in PBS overnight at 4°C, followed by rat anti-BrdU antibody (1:2000, AbD Serotec, UK) at 4°C for 48 h. BrdU-positive cells were visualized by incubating either with Cy5-conjugated donkey anti-rat IgG (H+L) (1:3000).

### Collagen IV, P4Hbeta, BRDU triple labeling

Sections were incubated first with rabbit polyclonal anti-collagen IV (1:1000, abcam, UK) followed by mouse anti-P4Hbeta monoclonal antibody (1:1000, Novus Biological, UK) followed by a mix of donkey anti-mouse Cy3 and donkey anti-rabbit Cy2 to visualize both primary antibodies. After a short fixation step with 4% paraformaldehyde, sections were subjected to the BrdU treatment and detection as above. BrdU was detected using donkey anti-rat Cy5.

### RECA, SMA, BRDU double and triple labeling

Sections were incubated first with a mix of rabbit polyclonal anti-actin (1:2000, Sigma, Munich, Germany) and mouse anti-rat endothelial cell antigen (RECA) (1:200, abcam, UK) followed by a mix of donkey-anti-rabbit-FITC 1:3000, and donkey-anti-mouse-rhodamine F(ab) 1:4000 both from Dianova, Hamburg, Germany. After a short fixation step with 4% paraformaldehyde, sections were subjected to the BrdU treatment and detection as above.

### BrdU, doublecortin double labeling

Cryostat, free-floating sections of 25 μm were fixed in 4% paraformaldehyde for 15 min and then washed extensively with PBS. After incubation in 50% Formamide/2X SSC for 2 h at 60°C, sections were washed again, first in 2x SSC and then in 10x PBS. After denaturization in 2N HCL at 37°C for 40 min, sections were made neutral by adding 0.1 M Borate buffer (pH 8.5). Thereafter sections were incubated with the guinea pig anti-doublecortin (DCX; Millipore) antibody overnight at 4°C followed by donkey anti-guinea pig IgG-biotin (Dianova, Hamburg, Germany) and streptavidin Alexa 488 (Life Technologies, Karslruhe, Germany). Finally, sections were incubated with rat anti-BrdU antibody (1:2000, AbD Serotec, Puchheim, Germany). BrdU-positive cells were visualized by incubating with Cy3-conjugated donkey anti-rat IgG (H+L) (1:3000).

### Cell quantitation

The number of labeled cells at the given reperfusion times was determined by counting cells on every tenth section in systematic random series across the entire region of interest. To this end, a sequence of confocal counting images of 161 × 242 × 25 μm, spaced 0.1 μm apart across a 25-μm-thick section and covering 30% of the infarcted area, was taken for fluorescently labeled cells. The resulting images were loaded into Image-J analysis software and the percentage of labeled cells was counted as previously described by us (14). Counting was done by two independent observers and the results are expressed as means ± SD.

### Quantitation of the number of remodelled blood vessels

Microvascular density was quantitated using the “hot spot” analysis covering 30% of the infarcted area. Briefly, hot-spots, i.e., regions with a high density of RECA/BrdU-positive blood vessels were identified using a 40 objective and were then counted using 20 objective, corresponding to a microscopic field of 0.7386 mm^2^ and the percentage of labeled blood vessels was calculated as previously described by us (10). Counting was done by two independent observers and the results are expressed as means ± SD.

### Microscopy

For light microscopy, an Axioscope A1 (Zeiss, Germany, Germany) was used. Confocal microscopy images were acquired using a Zeiss LSM710 laser-scanning confocal system with spectral detection capabilities, and Zen 2010 software version 6.0 (Carl Zeiss Microscopy GmbH, Jena, Germany) was used for image acquisition and analysis. Excitation light was provided by 488, 543, and 634 nm laser lines; fluorescence emission was detected at 500–530 nm for FITC (green), 550–600 nm for Rhodamin (red), and 650–710 nm for Cy5 (blue) in separate tracks, using a confocal aperture of 1 Airy unit. Some of the images were acquired as z-stacks and 3D reconstruction was performed using a software algorithm (maximal projection).

## Statistical analysis

The main effects of time and manipulation (stroke vs. sham), as well as interactions, were evaluated by ANOVA followed by Tukey *post-hoc* analyses using Prism software. The level of significance was set at *P* ≤ 0.05, two-tailed test.

## Results

### BRDU administration prior stroke had no effect on the infarct volume

The development of the infarct core was visualized by immunohistochemistry using anti NeuN antibodies, a sensitive indicator of neuronal viability (14). The development of the infarct was larger (129 ± 39 mm^3^) in in the first 3 days, presumably because of edema buildup (16), and stabilized at 116 ± 29 mm^3^ by day 28 (Figures 1C,D). However, the infarcted cortical volumes at 28 days were largely similar and independent of the BrdU treatment (Figures 1C,D).

### Brain vasculature is continuously remodelled in the adult non-injured brain

**Figure 2:**
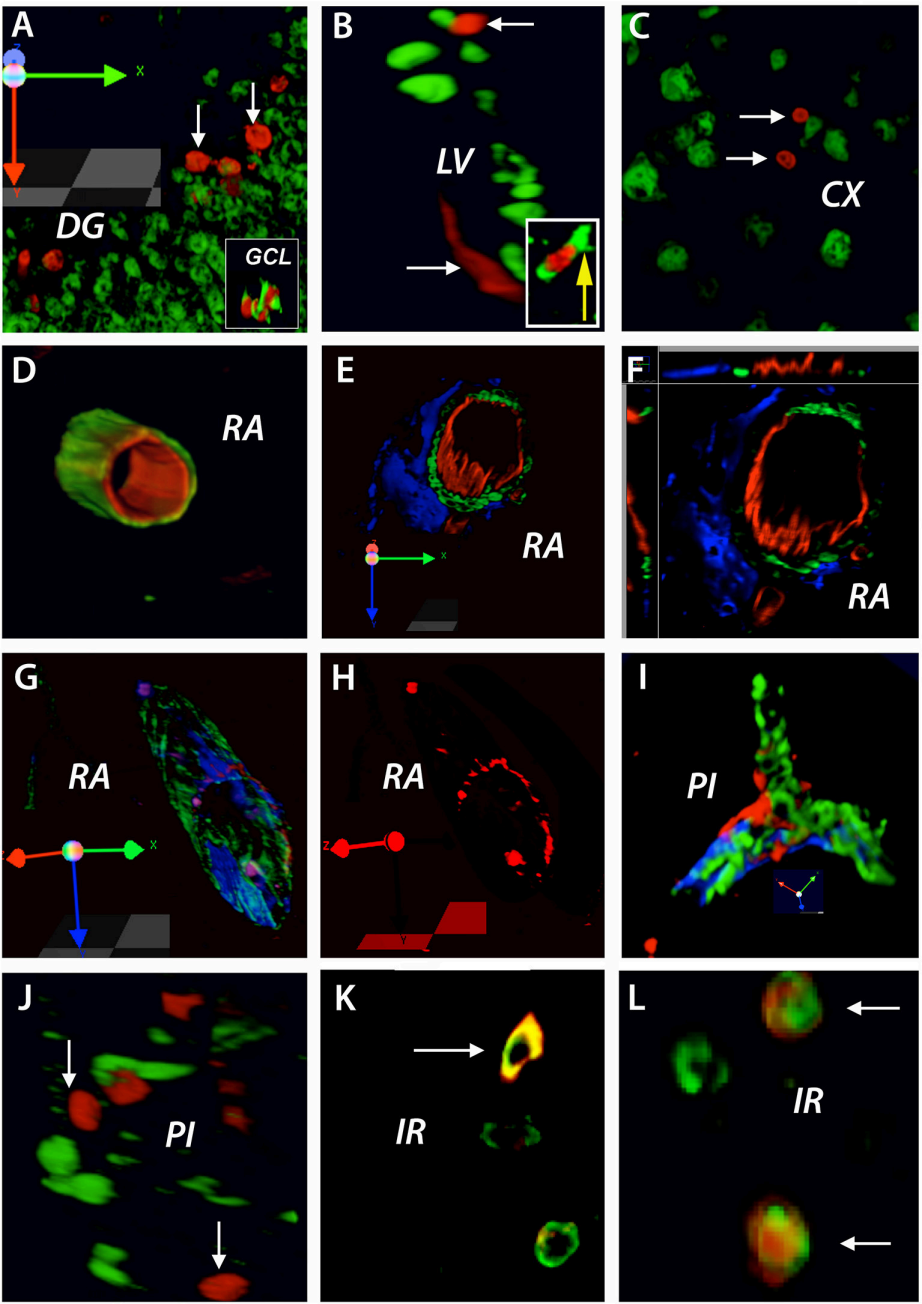
BrdU treatment before brain injury: brain vasculature is continuously remodeled in the adult non-injured brain. At 3 days after stroke, BrdU was incorporated preferentially into the nuclei of cells in the proximity of the dentate gyrus (DG) **(A)**. By double labeling these nuclei were identified as DCX neuronal precursor cells (**A**, inset). Likewise, BrdU/DCX co-localized cells were also found in the subventricular zone of the lateral ventricle (LV) (**B**, inset) as well as in the cortex (CX) of the non-injured tissue **(C)**. BrdU-positive nuclei co-localized with the marker of rat endothelial cells, RECA, lining the lumen of existing blood vessels in the rat cortex in remote areas (RA) relative to the infarct location **(D)**. By triple immunostaining, the co-localized BrdU/RECA cells were covered by smooth muscle actin-positive cells which were, most likely pericytes **(E,F)**. In a 3D image, the newly incorporated cells had a patchy distribution **(G,H)**. BrdU-positive cells having a patchy distribution were also found in branched blood vessels in the infarcted area **(I)**. However, BrdU-positive nuclei did not co-localized with proliferating microglia cells in the perilesional area **(J)**. Nevertheless, at 28 days post-stroke, co-localized BrdU/NeuN cells were found in the region beyond the inhibitory glial scar, “islet of regeneration, IR” **(K,L)**. The total number of animals at each time points was *N* = 10.

By injecting BrdU before brain injury we were able to identify proliferating cells like endothelial precursor cells and neuronal precursor cells in the non-injured adult rat brain that might be used in the brain response to cerebral ischemia. Using this method, we found that at 3 days after stroke, BrdU was incorporated preferentially into the nuclei of cells in the proximity of the dentate gyrus (DG) (Figure 2A). By double labeling these nuclei were identified as DCX neuronal precursor cells (Figure 2A, inset). Likewise, BrdU-positive nuclei that co-localized with DCX-positive cells were also found in the subventricular zone of the lateral ventricle (LV) (Figure 2B and inset) as well as in the cortex (CX) of the non-injured tissue (Figure 2C). Furthermore, BrdU-positive nuclei co-localized with the marker of the rat endothelial cells, RECA, lining the lumen of existing blood vessels in the rat cortex in remote areas (RA) relative to the infarct location (Figure 2D). By triple immunostaining, the co-localized BrdU/RECA cells were covered by smooth muscle actin-positive cells which were, most likely pericytes (Figures 2E,F). Interestingly, in a 3D image, the newly incorporated cells had a patchy distribution, suggesting a random insertion of endothelial cells into existing blood vessels (Figures 2G,H).

Quite surprisingly, BrdU-positive cells having a patchy distribution were also found in branched blood vessels in the infarcted area (Figure 2I). However, we could not detect BrdU-positive nuclei in proliferating microglia cells in the perilesional area (Figure 2J). Nevertheless, at 28 days post-stroke, we found co-localized BrdU/RECA cells (Figure 2K) as well as BrdU/NeuN cells (Figure 2L) in the region of soft tissue beyond the inhibitory glial scar, that we dubbed “islet of regeneration” because of presence of numerous blood vessels and endothelial cells (10).

### After stroke, BRDU-labeled nuclei are incorporated mostly in proliferating endothelial cells

**Figure 3:**
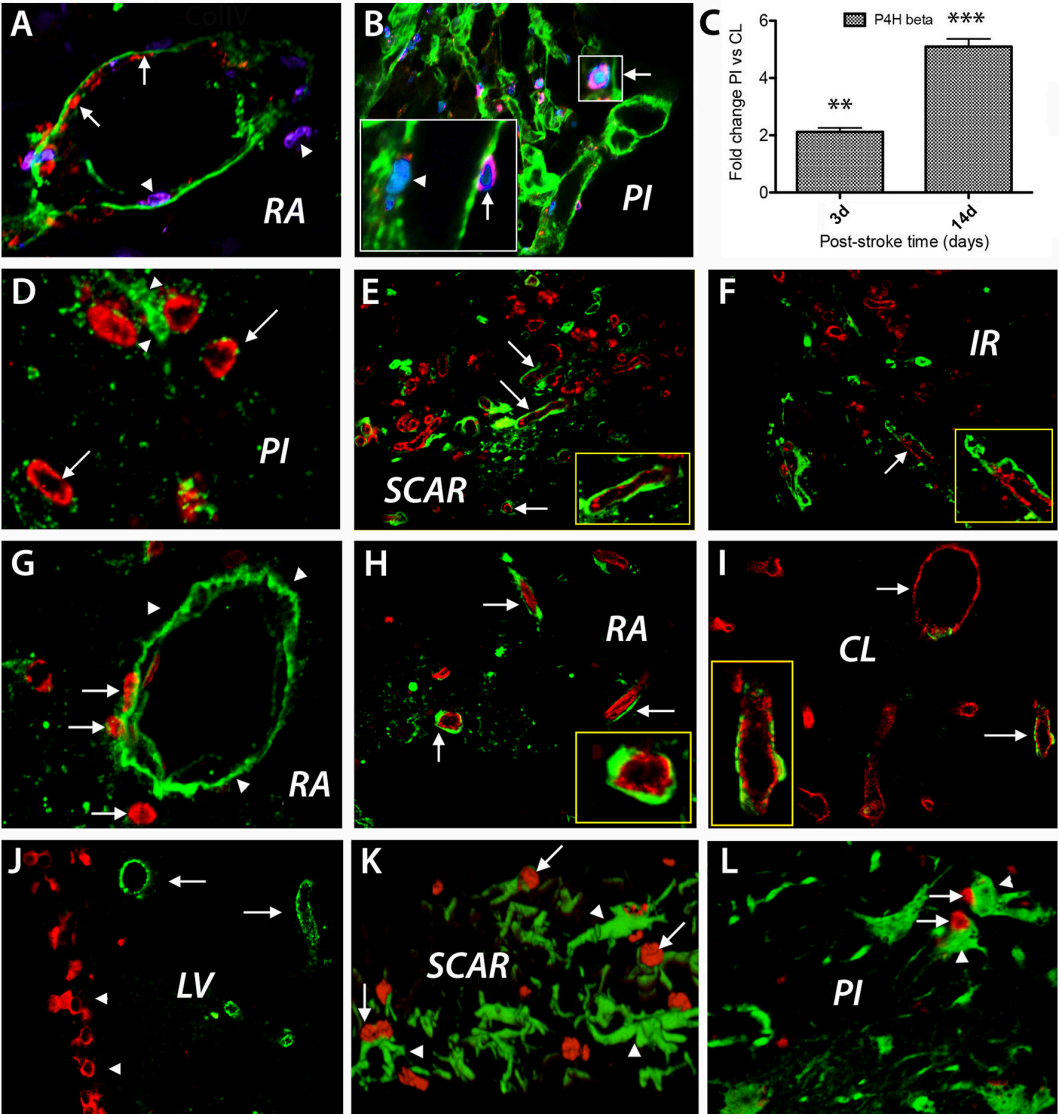
BrdU treatment after cerebral ischemia. Cell phenotyping. BrdU injection daily after focal ischemia led to labeling of endothelial cells lining blood vessel in remote area to the stroke lesion (**A**, red color, arrows) at day 3 after the ischemic event. Concurrently, cells which express the endothelial marker P4Hb that apparently cross large blood vessels (**B**, blue color, arrowheads) labeled with anti-collagen IV antibodies (green color) were detected. At day 14 post-stroke, proliferating endothelial cells were still detected using double labeling with anti-P4Hbeta (**B**, insets, red, arrows) and anti-BrdU antibodies (**B**, insets, blue, arrowhead, and insets). Using these markers, we could visualize endothelial cells detaching from the disintegrating blood vessels whose basement membrane was labeled with anti-collagen IV antibodies (**B**, green). The increased expression of *P4Hbeta* mRNA was confirmed by RT-PCR using P4Hbeta-specific primers at days 3 and 14 post-stroke when we expected an increase in *P4Hbeta* mRNA expression in endothelial cells in response to hypoxia **(C)**. By day28 after stroke, new blood vessels were emerging in the perinfarcted area, most of which were BrdU+ (**D**, red, arrows) and were, most likely, endothelial cells embedded in a laminin matrix (**D**, green, arrowheads). Double labeled BrdU+ (red) and RECA+ (green) blood vessels densely populated the scar region **(E)**. Double labeled BrdU+ (red) and RECA+ (green) blood vessels also emerged in the region beyond the glial scar, which we dubbed “islet of regeneration,” IR (**F**, arrow). Even at day 28 after the ischemic event BrdU+ cells seemingly leak through the wall of the blood vessels labeled with anti-laminin antibodies (**E**, red, arrows), into the vicinity of the infarcted area (**G**, red, arrows). Blood vessel double labeled for BrdU and RECA are still present in remote areas to the stroke lesion (**H**, arrows) and in the contralateral side (**I**, red, arrows). BrdU^+^ nuclei were also distributed in the “pinwheel” architecture of the ventricular (**J**, red) while the RECA^+^ cells occupied an adjacent, distal position (**I**, green). At day 3 after stroke, most of the neuroepithelial cells in the scar region and in the perilesional area displayed the nestin antigens (**K,L**; arrowheads). For immunohistochemistry the total number of animals at days 3 and 14 was *N* = 7. For RT-PCR the number of animals at days 3 and 14 was *N* = 3. The total number of animals at day 28 was *N* = 7. ^**^P = 0.01; ^***^P = 0.001.

Focal cerebral ischemia leads to blood vessel disintegration and endothelial cells proliferation (17). A good marker of proliferating endothelial cells in response to hypoxia, is prolyl 4-hydroxylase beta (18). Under hypoxic conditions, the proliferating endothelial cells express prolyl 4-hydroxylase (P4Hbeta), which catalyzes hydroxylation of the aminoacid proline in collagen fibrils. Injection of BrdU daily after cerebral ischemia led, at day 3 after the ischemic event, to labeling of endothelial cells lining blood vessels in remote area to the stroke lesion (Figure 3A, red color, arrows). Concurrently, we detected cells expressing the endothelial marker P4Hb that apparently crossed large blood vessels (Figure 3A, blue color, arrowheads) that were labeled with anti-collagen IV antibodies (Figure 3A, green color). The increased expression of *P4Hbeta* mRNA was confirmed by RT-PCR using P4Hbeta-specific primers at days 3 and 14 post-stroke when we expected an increase in *P4Hbeta* mRNA expression in endothelial cells in response to hypoxia (Figure 3C). At day 14 post-stroke, proliferating endothelial cells expressing P4Hbeta antigens were still present (Figure 3B and insets). Using these markers, we could visualize endothelial cells detaching from the disintegrating blood vessels those basement membrane was labeled with anti-collagen IV antibodies (Figure 3B, green).

By day 28 after stroke, new blood vessels were observed in the perilesional area, most of which were BrdU+ (Figure 3D, red, arrows) and were, most likely, endothelial cells embedded in a laminin matrix (Figure 3D, green, arrowheads). Most interestingly, double labeled BrdU+ (red) and RECA+ (green) blood vessels densely populated the scar region, which isolates the infarct area and is considered to inhibit axonal growth after stroke (Figure 3E). Further, double labeled BrdU+ (red) and RECA+ (green) blood vessels also emerged in the region beyond the glial scar (Figure 3F).

Quite unexpectedly, even at day 28 after the ischemic event, BrdU+ cells seemingly leaking through the wall of the blood vessels labeled with anti-laminin antibodies (Figure 3E, red, arrows), were present in the peri-infarcted area (Figure 3G, red, arrows). At the same time, blood vessel double labeled for BrdU and RECA were present in remote areas relative to the stroke lesion (Figure 3H, arrows) and in the contralateral side (Figure 3I, red, arrows).

In other remote areas to the infarct core like the lateral ventricle, the BrdU^+^ nuclei were distributed mainly in the “pinwheel” architecture of the ventricular epithelium (19, 20) (Figure 3J, BrdU^+^ nuclei shown in red) while the RECA^+^ cells occupied an adjacent, distal position (Figure 3J, RECA^+^ cells shown in green). Next, we investigated the presence of other proliferative BrdU-labeled cells in the perilesional area and found that at day 3 after stroke most of the proliferating cells displayed nestin antigens, strongly suggesting a neuroepithelial origin, both in the scar region and the perilesional area (Figures 3K,L; arrows).

## Discussion

Stroke induces a specific remodeling of the brain vasculature. However, the origin of the cells contributing to post-stroke cerebral blood vessel remodeling is poorly documented. By BrdU-labeling prior to stroke and immunohistochemistry we found that before injury there is a continuous remodeling of the adult brain vasculature by the incorporation of BrdU into the nuclei of endothelial cells lining the lumen of existing blood vessels in the adult rat cortex. Following injury, BrdU-positive cells were found in branched blood vessels in the infarcted area and in the “pinwheel” architecture of the ventricular epithelium. By BrdU-labeling after stroke and immunohistochemistry we found that BrdU/P4Hb co-labeled endothelial cells detaching from the disintegrating blood vessel at days 3–14 after the ischemic event. At 28 days post-stroke, we found co-localized BrdU/RECA and BrdU/NeuN cells in the region beyond the inhibitory glial scar, that we dubbed “islet of regeneration.” Other cells that incorporated BrdU+ nuclei were neuroepithelial cells and neuronal precursor cells.

### Brain vasculature remodeling in the adult non-injured brain

The adult brain vascular system is considered to be stable under normal physiological conditions (21). Therefore, data on brain vasculature remodeling in the adult brain is scarce. BrdU is widely used to label proliferating cells in the brain. BrdU incorporation into the nuclei of cells in the proximity of two neurogenic zones, the dentate gyrus and ependymal cell layer of the subventricular zone was therefore, not surprising (22). However, the incorporation of BrdU into the nuclei of endothelial cells lining the lumen of existing blood vessels in the rat cortex was rather surprising. Furthermore, the patchy distribution of endothelial cells with a BrdU nucleus highly suggests a random insertion of endothelial cells into existing blood vessels. As to the origin of these cells, they are most likely derived from endothelial progenitor cells (EPCs) which are normally present in the bone marrow and peripheral cells (23, 24). Likewise, the EPCs cells are most likely, at the origin of endothelial cells lining the newly formed blood vessels in the region beyond the inhibitory scar (24). Also unexpected was the incorporation of BrdU cells into nuclei of cells scattered into the non-injured cortex of the rat brain that probably entered the brain from the circulation via leptomeningeal blood vessels as previously reported by us (25).

### Brain vasculature remodeling in response to cerebral ischemia

After cerebral ischemia, a cascade of events that leads to vascular remodeling is triggered. One such process is arteriogenesis in response to the increased cerebral blood volume (CBV) after stroke (26, 27). Likewise, after transient MCA occlusion in rats, pial microcirculation remodeling along with new arterioles can be seen sprouting from the penumbra vessels and overlapping the ischemic core (28). Prolonged hypoxia induces blood vessel dilation, disintegration, pericytes detachment and migration, and EC proliferation. Further, when capillaries lose pericytes, they become hyperdilated and hemorrhagic leading to edema, which is the main cause of death in the first week after stroke and facilitates the infiltration of circulating, prior to stroke BrdU-labeled bone marrow derived endothelial precursor cells (24, 29).

BrdU injection shortly after stroke led to persistent labeling of numerous proliferating cells co-expressing BrdU and P4Hbeta, a marker of proliferating endothelial cells, emanating or detaching from the disintegrating blood vessels those basement membrane was labeled with anti-collagen IV antibodies as previously shown by us (17). However, it is hard to distinguish between bone marrow derived endothelial precursor cells and blood vessel wall-derived endothelial cells. Since shortly after BrdU injection blood vessels in remote areas to the infarct core and in the contralateral non-lesioned cortex showed co-labeled BrdU/RECA^+^ endothelial cells, we reasoned that these cells are derived from the bone marrow and reflect the normal remodeling of the cerebral vasculature.

In the damaged cortex, a BrdU/P4H double labeling in close proximity to collagen IV-labeled basement membrane suggests that, in addition to bone marrow-derived endothelial cells, the disintegrating vascular wall itself could be also a source of proliferating endothelial cells (17). At 4 weeks after stroke, new blood vessels were observed in the perilesional area, most of which were BrdU- and RECA-positive and were, most likely, endothelial cells embedded in a laminin matrix. Such cells are more likely bone marrow-derived endothelial cells that are present in the systemic circulation and leaked into the damaged area. Such cells are able to differentiate into endothelial cells in the perilesional area (30).

Most interestingly, double labeled BrdU+ (red) and RECA+ (green) blood vessels densely populated the scar region, which is considered by many neuroscience scholars as a region which is refractory to axonal growth and regenerative events, generally (Figure 3E). This finding suggests that the scar region which is made mostly of astroglial and fibroblasts-like cells, is actively maintained by a network of blood vessels. Further, the presence of double labeled BrdU+ and RECA+ blood vessels along with BrdU/NeuN-positive cells in the region which we dubbed “islet of regeneration,” beyond the glial scar, Buga et al. (10) suggests that vasculogenesis and neurogenesis may occur beyond the scarring region and do not need a “template.”

Quite unexpectedly, even at day 28 after the ischemic event BrdU+ cells seemingly leak through the basal lamina of the blood vessels the infarcted area and in remote areas to the stroke lesion suggesting that angiogenesis after stroke share similarities with angiogenesis in tumors i.e., the newly formed blood vessels are disorganized and leaky possibly via the STAT1/STAT3 pathway (10, 31).

In the non-lesioned subventricular zone, the BrdU^+^ nuclei were incorporated in the “pinwheel” architecture of the ventricular epithelium (19, 20) and did not overlap with the RECA^+^ cells. The significance of this finding is currently not known.

## Conclusions

**Figure 4:**
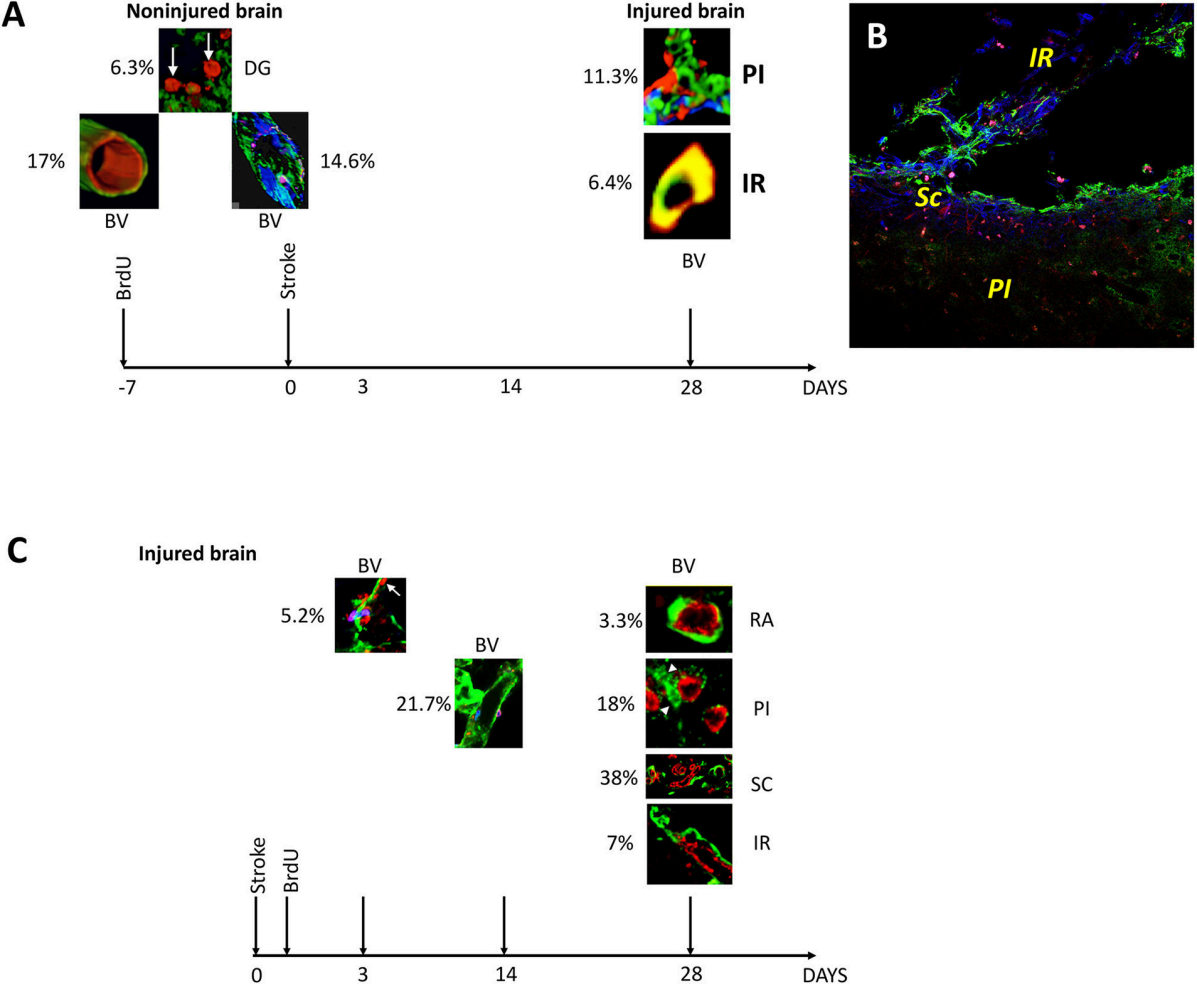
Overview and quantification of BrdU-labeled blood vessels in the non-injured and injured adult rat brain. **(A)** At 3 days after stroke, BrdU was incorporated preferentially into 6.3% of the neurons in the dentate gyrus (DG). BrdU-positive nuclei also co-localized with the marker of rat endothelial cells, RECA, in 17% of blood vessels in the rat cortex in remote areas (RA) relative to the infarct location. By triple immunostaining the newly incorporated cells had a patchy distribution in 14.6% of existing blood vessels. By day 28 after the stroke, BrdU-positive cells were also found in 11.3% of the branched blood vessels in the infarcted area and in 6.4% of the blood vessels in the region of soft tissue beyond the fibrotic scar **(B)**. **(C)** At day 3 post-stroke, prolyl 4-hydroxylase (P4Hbeta) was expressed in 5.2% of the endothelial cells detaching from the wall of the disintegrating blood vessels. Their number increased to 20.7% by day 14 after cerebral ischemia. By day 28 after stroke, new blood vessels were emerging in the perinfarcted area, 18% of which were BrdU+. More (38%) double labeled BrdU/RECA-positive blood vessels densely were found in the scar region while 7% double labeled BrdU/RECA-positive blood vessels were localized in the region beyond the glial scar, IR. At the same time, a small fraction (3.3%) of double labeled blood vessels were still present in remote areas relative to the stroke.

First, BrdU injection prior to stroke led to the patchy distribution of newly incorporated endothelial cells in mature blood vessels of the adult rat brain. Secondly, BrdU injection prior to stroke, specifically labeled neuronal precursors cells in the region beyond the inhibitory scar region which seems to be permissive to regenerative events. Third, BrdU injection after stroke led to the labeling of endothelial cells crossing or detaching from the disintegrating blood vessels, and their incorporation into new blood vessels in the infarct core, scar tissue and the region beyond. Fourth, BrdU injection after stroke led to the specific incorporation of BrdU^+^ nuclei into the “pinwheel” architecture of the ventricular epithelium. The pre- and post-stroke events of BrdU incorporation are summarized along with counting of immunofluorescently labeled cells and blood vessels, in Figure 4.

## Author contributions

RS: conception and design, animal surgery, RT-PCR and immunohisotchemistry on rat sections, manuscript writing; DG: conception and design, collection and/or assembly of data, data analysis and interpretation, manuscript writing; MP: animal perfusion, collection and/or assembly of data, data analysis, and interpretation; OM: collection and/or assembly of data, data analysis and interpretation; RE: collection and/or assembly of data, data analysis and interpretation, imaging analysis and administrative support; AP-W: conception and design, financial support, supervision of the project, data analysis and interpretation, and manuscript writing.

### Conflict of interest statement

The authors declare that the research was conducted in the absence of any commercial or financial relationships that could be construed as a potential conflict of interest.

## References

[1] Ruan L Wang B ZhuGe Q Jin K. Coupling of neurogenesis and angiogenesis after ischemic stroke. Brain Res. (2015) 1623:166–73. 10.1016/j.brainres.2015.02.042 25736182 PMC4552615

[2] Masuda H Asahara T. Post-natal endothelial progenitor cells for neovascularization in tissue regeneration. Cardiovasc Res. (2003) 58:390–8. 10.1016/S0008-6363(02)00785-X 12757873

[3] Hayashi T Deguchi K Nagotani S Zhang H Sehara Y Tsuchiya A. Cerebral ischemia and angiogenesis. Curr Neurovasc Res. (2006) 3:119–29. 10.2174/156720206776875902 16719795

[4] Caiado F Dias S. Endothelial progenitor cells and integrins: adhesive needs. Fibrogenesis Tissue Rep. (2012) 5:4. 10.1186/1755-1536-5-4 22410175 PMC3323425

[5] Liman TG Endres M. New vessels after stroke: postischemic neovascularization and regeneration. Cerebrovasc Dis. (2012) 33:492–9. 10.1159/000337155 22517438

[6] Yuewen Ma Lin Qiang Man He Exercise therapy augments the ischemia-induced proangiogenic state and results in sustained improvement after stroke. Int J Mol Sci. (2013) 14:8570–84. 10.3390/ijms14048570 23598418 PMC3645762

[7] Krupinski J Kaluza J Kumar P Kumar S Wang JM. Role of angiogenesis in patients with cerebral ischemic stroke. Stroke (1994) 25:1794–8. 10.1161/01.STR.25.9.1794 7521076

[8] Krupinski J Kumar P Kumar S Kaluza J. Increased expression of TGF-beta 1 in brain tissue after ischemic stroke in humans. Stroke (1996) 27:852–7. 10.1161/01.STR.27.5.852 8623105

[9] Krupinski J Issa R Bujny T Slevin M Kumar P Kumar S. A putative role for platelet-derived growth factor in angiogenesis and neuroprotection after ischemic stroke in humans. Stroke (1997) 28:564–73. 10.1161/01.STR.28.3.564 9056612

[10] Buga AM Margaritescu C Scholz CJ Radu E Zelenak C Popa-Wagner A. Transcriptomics of post-stroke angiogenesis in the aged brain. Front Aging Neurosci. (2014) 6:44. 10.3389/fnagi.2014.00044 24672479 PMC3957426

[11] Vikman P Edvinsson L. Gene expression profiling in the human middle cerebral artery after cerebral ischemia. Eur J Neurol. (2006) 13:1324–32. 10.1111/j.1468-1331.2006.01496.x 17116215

[12] Popa-Wagner A Schröder E Walker LC Kessler C. Beta-Amyloid precursor protein and ss-amyloid peptide immunoreactivity in the rat brain after middle cerebral artery occlusion: effect of age. Stroke (1998) 29:2196–202. 9756603 10.1161/01.str.29.10.2196

[13] Livak KJ Schmittgen TD. Analysis of relative gene expression data using real-time quantitative PCR and the 2(-Delta Delta C(T)) Method. Methods (2001) 25:402–8. 10.1006/meth.2001.1262 11846609

[14] Popa-Wagner A Badan I Walker L Groppa S Patrana N Kessler C. Accelerated infarct development, cytogenesis and apoptosis following transient cerebral ischemia in aged rats. Acta Neuropathol. (2007) 113:277–93. 10.1007/s00401-006-0164-7 17131130

[15] Popa-Wagner A Carmichael ST Kokaia Z Kessler C Walker LC. The response of the aged brain to stroke: too much, too soon? Curr Neurovasc Res. (2007) 4:216–27. 10.2174/156720207781387213 17691975

[16] Joseph C Buga AM Vintilescu R Balseanu AT Moldovan M Junker H. Prolonged gaseous hypothermia prevents the upregulation of phagocytosis-specific protein annexin 1 and causes low-amplitude EEG activity in the aged rat brain after cerebral ischemia. J Cereb Blood Flow Metab. (2012) 32:1632–42. 10.1038/jcbfm.2012.65 22617647 PMC3421103

[17] Popa-Wagner A Dinca I Yalikun S Walker L Kroemer H Kessler C. Accelerated delimitation of the infarct zone by capillary-derived nestin-positive cells in aged rats. Curr Neurovasc Res. (2006) 3:3–13. 10.2174/156720206775541732 16472121

[18] Dai Z Li M Wharton J Zhu MM Zhao YY. Prolyl-4 hydroxylase 2 (PHD2) deficiency in endothelial cells and hematopoietic cells induces obliterative vascular remodeling and severe pulmonary arterial hypertension in mice and humans through hypoxia-inducible factor-2α. Circulation (2016) 133:2447–58. 10.1161/CIRCULATIONAHA.116.021494 27143681 PMC4907810

[19] Liebner S Corada M Bangsow T Babbage J Taddei A Czupalla CJ. Wnt/beta-catenin signaling controls development of the blood-brain barrier. J Cell Biol. (2008) 183:409–17. 10.1083/jcb.200806024 18955553 PMC2575783

[20] Gajera CR Emich H Lioubinski O Christ A Beckervordersandforth-Bonk R Yoshikawa K. LRP2 in ependymal cells regulates BMP signaling in the adult neurogenic niche. J Cell Sci. (2010) 123(Pt 11):1922–30. 10.1242/jcs.065912 20460439

[21] Xiong Y Mahmood A Chopp M. Angiogenesis, neurogenesis and brain recovery of function following injury. Curr Opin Investig Drugs (2010) 11:298–308. 20178043 PMC2836170

[22] García-Verdugo JM Doetsch F Wichterle H Lim DA Alvarez-Buylla A. Architecture and cell types of the adult subventricular zone: in search of the stem cells. J Neurobiol. (1998) 36:234–48. 9712307 10.1002/(sici)1097-4695(199808)36:2<234::aid-neu10>3.0.co;2-e

[23] Guo X Liu L Zhang M Angela B Zhang J. Correlation of CD34+ cells with tissue angiogenesis after traumatic brain injury in a rat model. J Neurotrauma. (2009) 26:1337–44. 10.1089/neu.2008.0733 19226208 PMC2850294

[24] Zhang ZG Zhang L Jiang Q Chopp M. Bone marrow-derived endothelial progenitor cells participate in cerebral neovascularization after focal cerebral ischemia in the adult mouse. Circ Res. (2002) 90:284–88. 10.1161/hh0302.104460 11861416

[25] Buga AM Vintilescu R Balseanu AT Pop OT Streba C Tomescu E. Repeated PTZ treatment at 25-day intervals leads to a highly efficient accumulation of doublecortin in the dorsal hippocampus of rats. PLoS ONE (2012) 7:e39302. 10.1371/journal.pone.0039302 22768071 PMC3387140

[26] Liu J Wang Y Akamatsu Y Lee CC Stetler RA Lawton MT. Vascular remodeling after ischemic stroke: mechanisms and therapeutic potentials. Prog Neurobiol. (2014) 115:138–56. 24291532 10.1016/j.pneurobio.2013.11.004 PMC4295834

[27] Lake EMR Bazzigaluppi P Mester J Thomason LAM Janik R Brown M. Neurovascular unit remodelling in the subacute stage of stroke recovery. Neuroimage (2017) 146:869–82. 10.1016/j.neuroimage.2016.09.016 27664828

[28] Lapi D Colantuoni A. Remodeling of cerebral microcirculation after ischemia-reperfusion. J Vasc Res. (2015) 52:22–31. 10.1159/000381096 25896412

[29] Peichev M Naiyer AJ Pereira D Zhu Z Lane WJ Williams M. Expression of VEGFR-2 and AC133 by circulating human CD34(+) cells identifies a population of functional endothelial precursors. Blood (2000) 95:952–8. 10648408

[30] Mikirova NA Jackson JA Hunninghake R Julian K Kyle WHC Cathy A. Circulating endothelial progenitor cells: a new approach to anti-aging medicine. J Transl Med. (2009) 7:106. 10.1186/1479-5876-7-106 20003528 PMC2804590

[31] Wei LH Chou CH Chen MW Rose-John S Kuo ML Chen SU. The role of IL-6 trans-signaling in vascular leakage: implications for ovarian hyperstimulation syndrome in a murine model. J Clin Endocrinol Metab. (2013) 98:E472–84. 10.1210/jc.2012-3462 23348396

